# Placental abundances of IGF1, IGF2, IGFBP2, IGF2R, and PPARα are associated with birth weight

**DOI:** 10.1210/jendso/bvag130

**Published:** 2026-06-16

**Authors:** Felix Chelslín, Robert Kruse, Karolina Sollie, Lena Erlandsson, Yang Cao, Stefan R Hansson, Maria Lodefalk

**Affiliations:** Department of Pediatrics, School of Medical Sciences, Faculty of Medicine and Health, Örebro University, S-701 82 Örebro, Sweden; Department of Clinical Research Laboratory, School of Medical Sciences, Faculty of Medicine and Health, Örebro University, S-701 82 Örebro, Sweden; Inflammatory Response and Infection Susceptibility Centre (iRiSC), Faculty of Medicine and Health, Örebro University, S-701 82 Örebro, Sweden; Department of Clinical Research Laboratory, School of Medical Sciences, Faculty of Medicine and Health, Örebro University, S-701 82 Örebro, Sweden; Department of Obstetrics and Gynecology, Institute of Clinical Sciences Lund, Faculty of Medicine, Lund University, BMC I12, 221 84 Lund, Sweden; Skåne University Hospital, Lund/Malmö, SE-205 01 Malmö, Sweden; Clinical Epidemiology and Biostatistics, School of Medical Sciences, Faculty of Medicine and Health, Örebro University, S-701 82 Örebro, Sweden; Unit of Integrative Epidemiology, Institute of Environmental Medicine, Karolinska Institute, 171 77 Stockholm, Sweden; Department of Obstetrics and Gynecology, Institute of Clinical Sciences Lund, Faculty of Medicine, Lund University, BMC I12, 221 84 Lund, Sweden; Skåne University Hospital, Lund/Malmö, SE-205 01 Malmö, Sweden; Department of Pediatrics, School of Medical Sciences, Faculty of Medicine and Health, Örebro University, S-701 82 Örebro, Sweden; University Health Care Research Centre, Faculty of Medicine and Health, Örebro University, S-701 82 Örebro, Sweden

**Keywords:** cord blood, small for gestational age, large for gestational age, PCR, immunohistochemistry, ELISA

## Abstract

**Context:**

The insulin/insulin-like growth factor (IGF) system is a key regulator of fetal growth and placental nutrient regulation.

**Objective:**

The objective of this study was to investigate associated gene and protein expressions of the insulin/IGF, adiponectin, and peroxisome proliferator-activated receptor (PPAR) pathways in placenta and cord blood from small-, appropriate-, or large-for-gestational-age (SGA, AGA, LGA) infants and corresponding first-trimester maternal blood.

**Methods:**

A total of 60 SGA, 109 AGA, and 55 LGA term infants (birth weight z scores <−2, −2 to +2, and >2, respectively) were included. Placental mRNA expression of 22 genes was analyzed using RT-qPCR. Immunohistochemistry (IHC) was used to determine the protein localization and abundance of 5 differentially expressed genes in 78 representative placental samples. Cord blood and maternal blood concentrations of related proteins were analyzed by ELISA.

**Results:**

Placental gene expression of *IGF1*, *IGF2*, *IGBP2*, *IGF2R*, and *PPARA* was significantly lower in the SGA group. A moderate correlation in expression levels was observed among differentially expressed genes, except for *IGF2R*. In the SGA group, IHC staining for IGF1, IGF2, IGF2R, and PPARα was weaker, while IGFBP2 staining was stronger. In the cord blood, the IGF1 and IGF2 concentrations increased successively from the SGA group to the LGA group, whereas the IGFBP2 concentration decreased successively. No differences in maternal blood were observed between the groups.

**Conclusion:**

Placental IGF1, IGF2, and PPARα signaling are positively associated with birth weight, whereas that of IGFBP2 is negatively associated, possibly reflecting a shared upstream regulation by nutrition. Conversely, placental IGF2R signaling may aid in normalizing abnormal fetal growth.

Postnatal linear growth is a complex process that depends on genetic prerequisites, nutrition, and several endocrine factors, including thyroid hormones, the growth hormone (GH)/insulin-like growth factor (IGF) axis, and sex hormones. This statement also holds true in part for prenatal growth. Prenatally, however, the IGF system is predominantly regulated by maternal nutrition and placental function rather than by GH, and compared with IGF1, IGF2 appears to be more important [1, 2]. The placenta transports nutrients and oxygen from the mother to the growing fetus, regulates maternal–fetal physiology during pregnancy, and stimulates fetal growth through the production and secretion of hormones [1, 2].

The insulin/IGF system is crucial for both fetal and placental growth. The insulin receptor and the IGF1 receptor (IGF1R) promote fetal growth, whereas the IGF2 receptor (IGF2R) inhibits growth by decreasing IGF2 bioavailability [1, 3]. The IGF binding proteins 1 to 7 (IGFBP1-7) also regulate the availability of IGFs, and variations in their levels in the placenta and umbilical cord blood have been associated with different birth weight outcomes [1, 3-6]. Similarly, differential adiponectin levels in maternal and umbilical cord blood have been associated with aberrant fetal growth [7-10], which is potentially related to the insulin-sensitizing effect of adiponectin [7]. Whether the placenta secretes adiponectin remains debated. For example, Lappas et al [8] reported adiponectin release from placental tissue, whereas Corbetta et al [9] were unable to detect any measurable levels of the protein in the placenta.

Another important nutrient-associated signaling pathway that may influence fetal growth is the peroxisome proliferator-activated receptor (PPAR) system. These receptors are transcription factors composed of 3 isoforms (PPARα, PPARγ, and PPARβ/δ). Their primary ligands consist of various kinds of fatty acids. After ligand binding, PPARs bind to DNA sequences termed PPAR-responsive elements (PPREs) together with their cofactors, the retinoid X receptors (RXRs), which initiate or repress transcription of target genes, influencing various metabolic processes [10, 11]. Clinically, PPARγ activation by glitazones and PPARα activation by fibrates have been used to reduce insulin resistance and dyslipidemia, respectively [12]. Peroxisome proliferator-activated receptors have also been proposed to regulate reproductive functions [13-15]. Placental PPARγ expression is positively associated with placental weight and birth weight, and downregulation of PPARγ expression has been reported in placentas of small-for-gestational-age (SGA) infants [14, 15].

Evidence from animal and in vitro studies suggests that the complex regulation of fetal growth relies on these intertwined signaling systems. For example, adiponectin decreases insulin signaling in human trophoblasts by activating PPARα signaling [16].

Both impaired and exaggerated fetal growth profoundly increase the risk of perinatal and neonatal morbidity and mortality and imply an increased long-term risk [17-22]. Therefore, the knowledge about the regulation behind fetal growth needs to increase. Previous studies have been limited by small sample sets or few investigated subjects [23].

This study aimed to investigate placental gene and protein expression as well as cord blood concentrations of various factors involved in the insulin/IGF, adiponectin, and PPAR signaling systems in a large study sample of SGA, appropriate-for-gestational-age (AGA), or large-for-gestational-age (LGA) neonates to elucidate the complex regulation of human fetal growth. Maternal first-trimester blood was also examined for potential biomarkers of aberrant fetal growth.

## Materials and methods

### Study design

This project was conducted in accordance with the Declaration of Helsinki and approved by the Regional Ethics Board, Uppsala, Sweden (2010/189/1). Written informed consent was obtained from all the participating women.

A stratified observational study design was used. The cases were divided into 2 groups: SGA and LGA infants. First, the differential placental expression (DE) of 22 genes of interest was investigated across the whole study sample (n = 224). The protein abundances of the DE genes were subsequently validated by immunohistochemistry (IHC) in placental tissues from a subset of infants (n = 79), which was selected to represent a polarized sample based on birth weights, leading to the inclusion of 26 SGA infants with the lowest birth weight z scores, 27 LGA infants with the highest birth weight z scores, and 26 AGA infants with birth weight z scores closest to 0. Serum concentrations of relevant proteins were measured in all available cord blood samples (n = 207). Due to limited resources, serum protein concentrations were only measured in mothers included in the IHC study and when available (n = 77).

### Inclusion criteria and demographic data

Placental and cord blood samples were obtained from a research sample collection at Örebro University Hospital, Örebro, Sweden. The samples were collected at the Department of Women's Health at the hospital between 2004 and 2012. Maternal serum samples from the first trimester were collected from clinical samples, and clinical data were obtained from medical records at antenatal care units and the Department of Women's Health at the hospital. The z scores for birth weight, length, and head circumference were calculated using Swedish reference data accounting for sex and gestational age [24]. The inclusion criteria were nonsmoking women aged 18 to 42 years, an early pregnancy body mass index (BMI) of 18.5 to 29.9 kg/m^2^, and singleton pregnancies with term (gestational age 37 + 0-41 + 6) vaginal delivery. The exclusion criteria included maternal chronic and gestational disease, infant asphyxia (defined as an Apgar score of <7 at 5 minutes), newborn chromosomal abnormalities, and anatomical malformations.

### Sample size calculation

A priori power analysis was conducted to estimate the sample size on the basis of previously reported gene expression levels of *PPARG*, *IGF1*, and *IGF2* [14, 25]. With a significance criterion of α = .05, power = 0.80, an effect size of ≥2, and a 1:2 allocation ratio to the study groups, the minimum sample sizes for the LGA and AGA groups were 52 and 104, respectively. A total of 55 LGA infants (defined as those whose birth weight z score was >2 [26]) were identified in the sample collection, fulfilling both the inclusion and exclusion criteria. For each LGA infant, 2 AGA infants (defined as those whose birth weight z scores were within ±2) were included and matched for fetal sex, maternal parity, early pregnancy BMI, age, and calendar date at the beginning of the pregnancy, in decreasing priority order. After the initial study design was completed, SGA infants (defined as those whose birth weight z score was <−2 [27]) were also included to allow a more comprehensive evaluation across the full spectrum of fetal growth. Small-for-gestational-age infants available for sample collection who met all the inclusion and exclusion criteria were included. Because of the limited number of SGA infants, they were not matched with the other included infants. In total, 55 LGA, 60 SGA, and 109 AGA infants were included.

### Sample collection

Immediately after delivery, the amniotic membranes were removed, and placental tissue samples measuring approximately 1 cm^3^ were collected from the maternal side, as previously described [28]. The marginal portion and areas near the cord of the placenta were avoided. The collected tissues were rinsed in 30 mL cold phosphate-buffered saline (PBS) (Gibco, Life Technologies, Stockholm, Sweden) and stored in 3 mL RNALater (Ambion, Stockholm, Sweden) at −80 °C until further analysis.

Maternal first-trimester blood samples were collected in 5 mL clot activator tubes (BD Vacutainer, Plymouth, United Kingdom) and centrifuged at 2400 × g for 7 minutes at room temperature. The maternal serum was stored at −20 °C. Umbilical cord venous blood samples were collected in 10 mL clot activator tubes (BD Vacutainer) and centrifuged at 2000 × g for 7 minutes at room temperature. The cord serum was stored at −80 °C.

### Real-time quantitative polymerase chain reaction

Approximately 10 mg placental tissue was cut from each sample, thawed, and used for RNA extraction with an Allprep DNA/RNA/protein kit (Qiagen, Nordic Sollentuna, Sweden; Catalog #80004) at the Clinical Research Laboratory at Örebro University Hospital. The extracted RNA was stored at −80 °C until further analysis. Tapestation 4150 (Agilent, Santa Clara, California, United States, RRID:SCR_019393) and RNA ScreenTape (Agilent, Catalog #5067-5576) were used to assess the RNA integrity number (RIN) for each extracted RNA sample. For cDNA synthesis, RNA was diluted to 200 ng/μL in molecular biology-grade, nuclease-free water (Thermo Fisher Scientific, Waltham, Massachusetts, United States; Catalog #R0582). Two cDNA synthesis reactions were performed for each sample, and the resulting products were pooled and further diluted.

The following genes of interest were analyzed: *IGF1*, *IGF2*, *IGF1R*, *IGF2R*, *INSR*, *IGFBP1*, *IGFBP2*, *IGFBP3*, *IGFBP4*, *IGFBP5*, *IGFBP6*, *IGFBP7*, *PPARA*, *PPARG*, *PPARD*, *RXRA*, *RXRB*, *ADIPOQ*, *ADIPOR1*, *ADIPOR2*, *APPL1*, and *APPL2*. Previously published next-generation sequencing (NGS) gene expression data were used to estimate the approximate expression level of each gene in human placental tissue. Genes with expected similar expression levels in the samples were combined into pairs for multiplex real-time quantitative polymerase chain reaction (RT-qPCR) analysis, after which assay validation was performed. The expression levels of the gene pairs were evaluated against a 7-point standard curve according to the manufacturer's recommendation using synthetic DNA (gBlocks from Integrated DNA Technologies, Coralville, IA, United States) as the template. The genes were analyzed in pairs using RT-qPCR, as shown in Table S1 [29]. However, the genes *IGF1R*, *IGFBP3*, *ADIPOQ*, and *APPL1* were evaluated individually.

Real-time quantitative polymerase chain reaction was performed using a Life Technologies QuantStudio 7 Real Time PCR System (Applied Biosystems, Waltham, Massachusetts, United States; RRID:SCR_020245) according to the manufacturer's instructions. Each assay was performed on separate 384-well plates prepared from the same batch of master mix and run in consecutive order. All the samples were run in duplicate. Subsequent RT-qPCR was performed for samples showing high technical variation (replicate SD > 0.167, as suggested by the manufacturer). Polymerase chain reaction reagents and plates were obtained from Thermo Fisher Scientific (Table S2 [30]). Gene expression was normalized to the geometric mean expression of the reference genes *CYC1* and *TOP1*, as previously described [31]. Cycle threshold (Ct) values > 35 or “not detected” were considered negative, according to the manufacturer's recommendations. All calculations were based on the ΔCt and ΔΔCt methods [32].

### Immunohistochemistry

A methodological evaluation was performed on separately collected placental tissues to optimize IHC using tissues preserved in RNALater. These new placenta samples were collected from 5 individual placentas at delivery. Each placental sample was divided into 2 adjacent samples, with one stored in RNALater (n = 5) and the other snap frozen (n = 5). Paraffin embedding was performed as described below. They were stained in parallel to identify the proteins of interest, and the staining results were evaluated by a pathologist and deemed comparable.

The placental tissue samples included in this study were thawed, rinsed in distilled H_2_O, and fixed in 4% formaldehyde at room temperature. The samples were dehydrated and embedded in paraffin. One sample from an LGA infant was excluded because of insufficient embedding. The samples were cut into 4-µm sections using an Epredia HM 355S Automatic Microtome (Epredia, New Hampshire, United States; RRID:SCR_026146) and mounted on a SuperFrost glass (Epredia, New Hampshire, United States). The sections were dried at 60 °C for 1 hour before deparaffinization and rehydration. The tissue slides were placed in a pressure cooker in 1:20 Diva decloaker solution (Biocare Medical, Concorde, United States) at 110 °C for 10 minutes to retrieve heat-induced epitopes. Immunohistochemical analysis was performed using an IntelliPATH FLX instrument (Biocare Medical). The tissue slides were subsequently washed with PBS containing 0.1% Tween (Biocare Medical). Background peroxidase activity was blocked by incubating the tissue sections with Peroxidazed 1 (Biocare Medical) for 5 minutes. Nonspecific background staining of the tissue sections was blocked with Background Sniper (Biocare Medical) for 15 minutes. The sections were stained with antibodies (Table S3 [33]) against proteins of interest for 1 hour in a moist chamber; GLUT1 expression served as a positive control. The antibodies were diluted with Da Vinci Green (Biocare Medical). Tissue sections were stained with anti-GLUT1 and were incubated using a mouse probe for 15 minutes. All tissue sections were incubated with an HRP-polymer for 30 minutes. Chromogen 3,3′-diaminobenzidine (DAB) substrate solution was added to tissue sections and incubated for 5 minutes. The sections were counterstained with hematoxylin (Histolab Products AB, Askim, Sweden) for 5 minutes. The slides were mounted with a Pertex mounting medium (Histolab Products AB) and scanned with a 3DHISTECH Pannoramic 250 Flash III slide scanner (3DHistech, Budapest, Hungary; RRID:SCR_022184). Imaging analysis was performed using a tissue section imaging system (CaseViewer version 2.1.2.69595; 3DHistech; RRID:SCR_017654). One blinded assessor (FC) evaluated all the samples at 40× magnification to determine the predominant location and intensity of staining within the tissues. Staining intensity was graded on a scale of 1 to 3 (weak, moderate, or strong), and positive staining rates were calculated as the number of stained cells divided by the total number of cells in each section studied.

### Analysis via ELISA

Cord serum and maternal serum concentrations of proteins associated with DE genes and expected to be found in the circulation (IGF1, IGF2, IGFBP2, and sIGF2R) were assessed by ELISA using a BioTek Epoch 2 Microplate Spectrophotometer (BioTek, Winooski, VT, United States; RRID:SCR_019740). The proteins were run in duplicate, with aliquots randomly assigned to separate plates. Details can be found in Table S4 [34].

### Statistical analysis

Data are presented as medians (minimum–maximum) for continuous variables or as absolute numbers (percentages) for categorical variables. The normality of continuous variables was assessed using the Shapiro–Wilk test. As most variables were nonnormally distributed, nonparametric methods were applied. Comparisons of continuous variables among the 3 study groups (SGA, AGA, and LGA) were performed using the Kruskal‒Wallis test. When a statistically significant overall difference was detected, pairwise comparisons were performed using the Wilcoxon rank-sum test. To account for multiple pairwise comparisons, *P* values were adjusted using the Benjamini‒Hochberg method. Categorical variables were compared using Pearson's chi-square test or the Fisher–Freeman–Halton exact test as appropriate. For analyses involving multiple genes, *P* values were adjusted for multiple testing using the Benjamini‒Hochberg method, with a predefined false discovery rate (FDR) threshold of 10%. Principal component analysis (PCA) was performed on the gene expression data to explore multivariable patterns and clustering among the study groups. Spearman's rank correlation test was used to analyze associations between gene expression, cord blood protein expression, and infant characteristics. To evaluate the proportion of variation in birth weight, birth length, and head circumference explained by biological and clinical variables, multiple linear regression analysis was employed, with each anthropometric measure used as a dependent variable. Independent variables included placental gene expression, cord blood protein expression, and clinical variables (sex, gestational age at birth, and maternal weight, height, and parity). Multicollinearity among independent variables was assessed using the variance inflation factor (VIF). Variables with a VIF > 5 were excluded from the final models. Statistical significance was set at a 2-sided *P* < .05. Statistical analysis was performed in R Project for Statistical Computing (version 4.4.2; R Core Team, 2024; RRID:SCR_001905) using the following packages: corrr, ggcorrplot, FactoMineR, factoextra, and dplyr.

## Results

### Clinical characteristics of the study sample

The clinical characteristics of all the women and infants included in the study are presented in Table 1. Women in the SGA group were more often nulliparous (*P* = .012) and had lower weights (*P* < .001) and shorter heights (*P* < .001) than those in the AGA and LGA groups were. They also had lower BMIs in early pregnancy than women in the LGA group did (*P* = .015). Additionally, delivery was more rarely induced among women in the AGA group than among the other women (*P* = .016).

**Table 1:** Maternal and infant characteristics in the total study sample and the subset used for deeper analyses

	Total study sample				Subjects included in IHC and maternal serum analyses			
	SGA N = 60	AGA N = 109	LGA N = 55	*P* value	SGA N = 26	AGA N = 26	LGA N = 27	*P* value
	Maternal characteristics							
Age (years)	28.6 (20.9-39.9)	31.2 (19.1-41.2)	31.8 (22.1-41.0)	.003	28.8 (22.5-37.3)	30.0 (19.1-35.7)	31.6 (22.1-39.5)	.127
Nulliparous (%)	37 (61.7%)	23 (21.1%)	9 (16.4%)	<.001	16 (61.5%)	7 (26.9%)	7 (26.9%)	.012
Early pregnancy weight (kg)	61.5 (49.0-90.0)	66.0 (48.0-89.0)	72.0 (55.0-90.0)	<.001	63.0 (49.0-74.0)	66.0 (48.0-86.0)	73.0 (62.0-90.0)	<.001
Height (cm)	164 (151-176)	168 (153-179)	169 (158-180)	<.001	162 (151-176)	168 (155-179)	171 (158-179)	<.001
Early pregnancy BMI (kg/m^2^)	22.6 (18.6-29.1)	23.7 (19.7-29.7)	24.9 (19.7-29.8)	.010	22.6 (19.8-28.9)	24.0 (19.7-29.4)	25.0 (21.1-29.1)	.031
	Delivery characteristics							
Labor induction (%)	17 (28.3%)	7 (6.4%)	10 (18.2%)	<.001	10 (38.5%)	2 (7.7%)	2 (7.7%)	.016
Vacuum extraction (%)	5 (8.3%)	2 (1.8%)	4 (7.3%)	.112	1 (3.8%)	0 (0%)	4 (15.4%)	.062
	Infant characteristics							
Gestational age (weeks)	40.1 (37.1-41.9)	40.0 (37.0-41.9)	39.6 (37.1-41.9)	.022	39.8 (37.1-41.9)	39.7 (37.6-41.9)	39.5 (37.1-41.9)	.343
Sex, number of males (%)	23 (38.3%)	52 (47.7%)	29 (47.3%)	.282	16 (61.5%)	14 (53.8%)	15 (57.7%)	.854
Birth weight (g)	2750 (2210-3200)	3685 (2720-4940)	4725 (4120-5560)	<.001	2660 (2210-3115)	3590 (3130-4070)	4810 (4120-5560)	<.001
Birth length (cm)	48 (44-51)	51 (46-55)	52 (49-55)	<.001	47 (44-50)	51 (48-55)	53 (49-55)	<.001
Head circumference (cm)	34 (32-37)	35 (32-39)	37 (33-40)	<.001	34 (32-37)	35 (32-39)	37 (33-40)	<.001
Birth weight z score	−2.38 (−3.61 – −2.01)	0.13 (−1.80-1.99)	2.18 (2.01-3.85)	<.001	−2.54 (−3.61 – −2.24)	0.02 (−0.37-0.36)	2.39 (2.13-3.85)	<.001
Birth length z score	−2.47 (−4.50 – −0.97)	−0.45 (−2.67-1.68)	1.13 (−0.68-2.91)	<.001	−2.55 (−4.50 – −1.61)	−0.35 (−1.42-1.68)	1.26 (−0.68-2.91)	<.001
Head circumference z score	−1.39 (−3.56-0.75)	−0.25 (−2.30-2.13)	1.02 (−1.17-3.29)	<.001	−1.52 (−2.53-0.75)	0.03 (−1.82-2.13)	1.03 (−1.17-3.29)	<.001

Data are medians (min–max) or numbers (%).

Abbreviations: AGA, appropriate for gestational age; BMI, body mass index; IHC, immunohistochemistry; LGA, large for gestational age; SGA, small for gestational age.

### Placental gene expression

**Figure 1:**
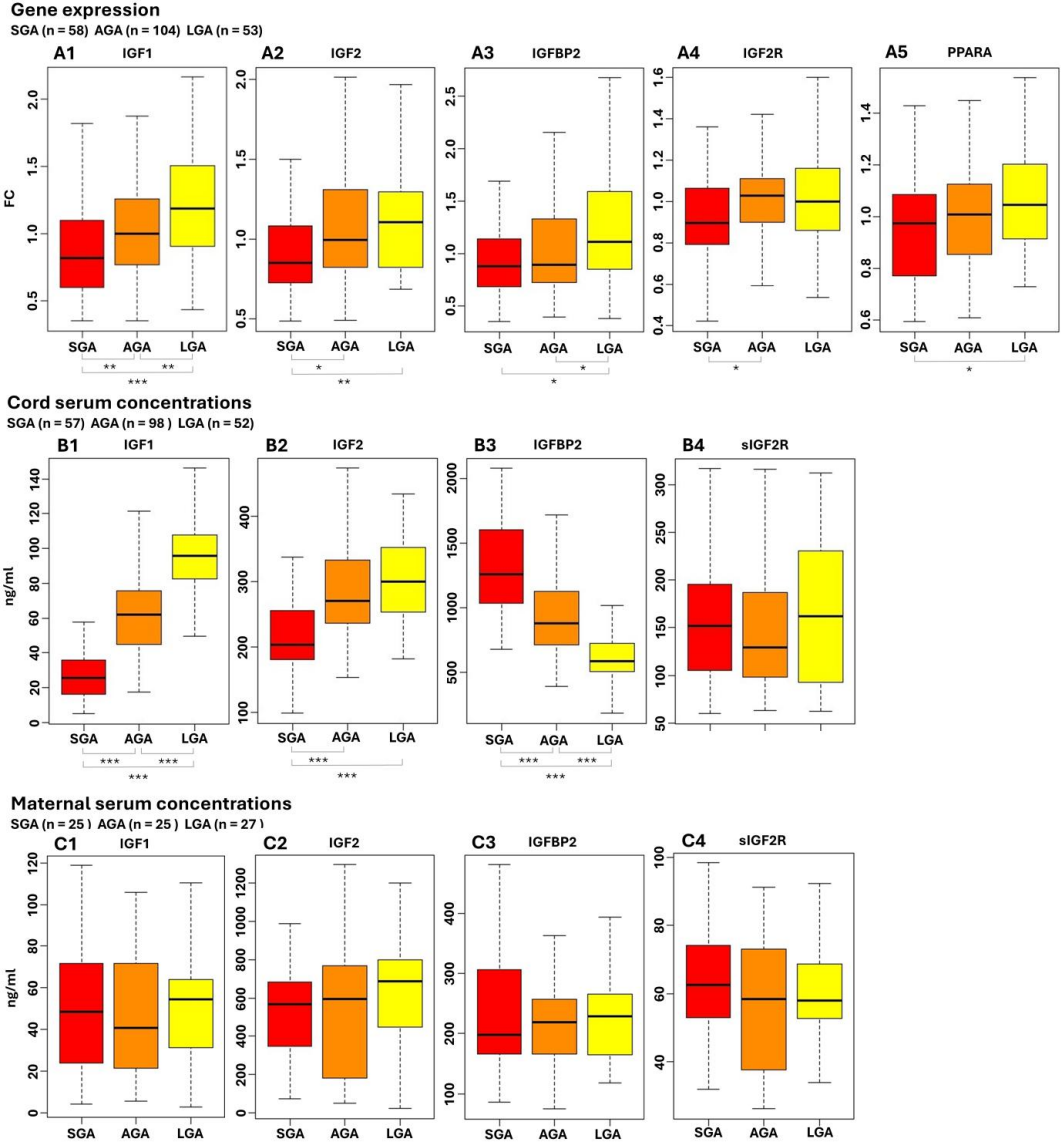
Gene expression levels of *IGF1*, *IGF2*, *IGFBP2*, *IGF2R*, and *PPARA* in term placental tissues (A1-A5) and protein concentrations in cord serum (B1-B4) and maternal first-trimester serum (C1-C4). The boxes represent the 25th and 75th percentiles, and the horizontal lines represent the median value and the upper (97.5th) and lower (2.5th) limits, respectively. Outliers are excluded. * = *P* < .05. ** = *P* < .01. *** = *P* < .001. Abbreviations: AGA, appropriate for gestational age; FC = fold change; the ratio of expression related to the mean expression in the AGA group; LGA, large for gestational age; SGA, small for gestational age.

The median RIN value for all extracted RNA samples was 8.4 (4.5-9.2). Nine RNA samples (from 2 SGA, 5 AGA, and 2 LGA infants) had a RIN < 7 and were excluded from the statistical analysis of gene expression. In 81% of the samples, the Ct value for *ADIPOQ* was >35; therefore, the expression of this gene was considered below the limit of detection and excluded from further analysis. Satisfactory expression was observed for the remaining 21 genes. Principal component analysis did not reveal any differences among the 3 groups (Fig. S1 [35]). However, univariable analyses, corrected for multiple comparisons, revealed that the gene expression of *IGF1*, *IGF2*, *IGFBP2*, *IGF2R*, and *PPARA* differed significantly among the groups considering the predefined FDR of 10%, even though the fold changes were small (Table 2). The expression levels of the following genes were significantly lower in the SGA group than in the LGA group: *IGF1* (*P* < .001), *IGF2* (*P* = .002), *IGFBP2* (*P* = .024), and *PPARA* (*P* = .012). The gene expression of *IGF1*, *IGF2*, and *IGF2R* was also significantly lower in the SGA group than in the AGA group (*P* = .003, *P* = .008, and *P* = .018, respectively). The gene expression of *IGF1* and *IGFBP2* was significantly lower in the AGA group than in the LGA group (*P* = .007 and *P* = .033, respectively) (Fig. 1, A1-A5).

**Table 2:** Comparisons of placental expression of genes of interest between term infants born small, appropriate, or large for gestational age, sorted by *P* value adjusted for multiple comparisons

Gene name	FC SGA	FC LGA	Adjusted *P* value
*IGF1*	0.8	1.2	.021
*IGF2*	0.9	1.1	.021
*IGFBP2*	1.0	1.1	.088
*PPARA*	0.9	1.1	.088
*IGF2R*	0.9	1.0	.088
*IGF1R*	0.9	1.0	.176
*RXRA*	1.0	0.9	.176
*IGFBP7*	0.9	1.1	.176
*IGFBP1*	1.0	1.0	.243
*APPL1*	0.9	0.9	.290
*ADIPOR1*	1.0	1.0	.334
*PPARD*	1.0	1.0	.334
*IGFBP3*	0.8	0.9	.367
*IGFBP5*	0.9	1.0	.476
*IGFBP6*	0.8	0.8	.507
*APPL2*	1.0	1.0	.583
*INSR*	0.9	1.0	.779
*IGFBP4*	1.0	1.0	.868
*PPARG*	1.0	1.0	.922
*RXRB*	1.0	1.0	.922
*ADIPOR2*	1.0	1.0	.944

Comparisons of gene expression among the 3 study groups were performed using the Kruskal‒Wallis test with the Benjamini‒Hochberg method for multiple comparisons.

Abbreviations: FC, fold change in relation to the expression in the group of appropriate-for-gestational age children; LGA, large for gestational age; SGA, small for gestational age.

### Placental immunohistochemistry

**Figure 2:**
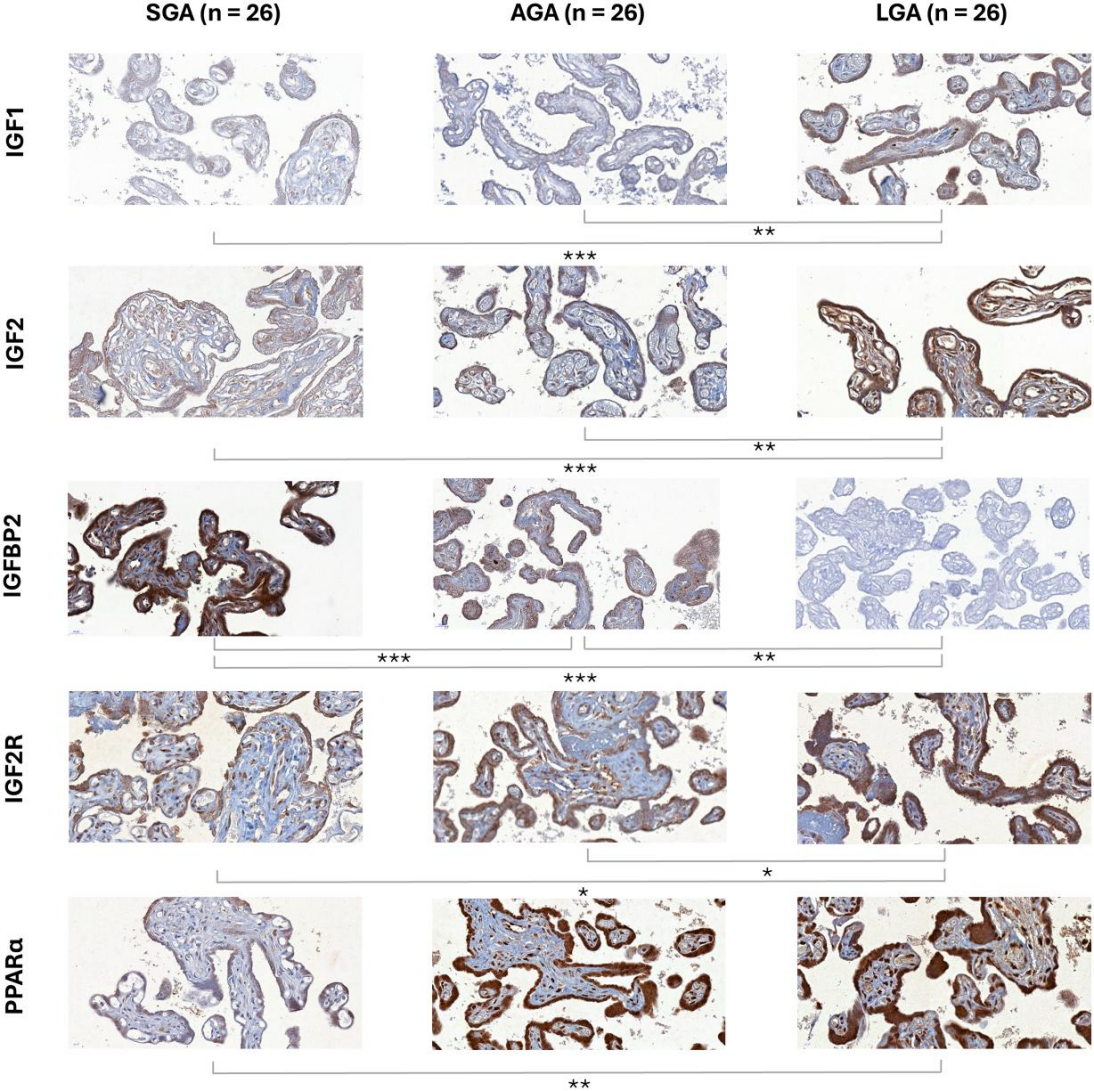
Images highlighting differences in immunohistochemical staining intensities for IGF1, IGF2, IGFBP2, IGF2R, and PPARα in placental tissues from small-, appropriate-, and large-for-gestational-age infants. Abbreviations: AGA, appropriate for gestational age; LGA, large for gestational age; SGA, small for gestational age. * = *P* < .05. ** = *P* < .01. *** = *P* < .001.

The IHC results revealed protein staining intensity differences among the groups, reflecting the gene expression levels except for IGFBP2. Strong expression of all the proteins studied was observed in both syncytiotrophoblasts and cytotrophoblasts within the placental villi. Strong staining was also observed in the cellular membranes for all the proteins, except for PPARα, for which staining was more prominent in the nucleus. No differences in the staining patterns of different placental cell types were detected between the study groups. No specific expression was detected in stromal cells or the vascular endothelium. Weaker staining of IGF1, IGF2, IGF2R, and PPARα was observed in the SGA group than in the LGA group (*P* = .001, *P* = .001, *P* = .010, and *P* = .004, respectively). Weaker staining was also observed for IGF1, IGF2, and IGF2R expression in the AGA group than in the LGA group (*P* = .001, *P* = .002, and *P* = .020, respectively). Stronger staining of IGFBP2 expression was observed in the SGA group than in the AGA and LGA groups (*P* < .001 and *P* < .001, respectively) and in the AGA group than in the LGA group (*P* = .003) (Fig. 2).

### ELISA results

Cord blood serum levels of IGF1, IGF2, and IGFBP2 varied among the groups (Fig. 1, B1-B4). Higher levels of IGF1 and IGF2 and lower levels of IGFBP2 were observed in the cord blood serum samples of the SGA group than in those of the AGA and LGA groups (*P* < .001 for all comparisons). Higher serum levels of IGF1 and lower IGFBP2 levels were also observed in the AGA group cord blood serum samples than in those of the LGA group (*P* < .001 and *P* < .001, respectively). No differences in the cord serum sIGF2R levels were detected between the study groups. The IGF1, IGF2, IGFBP2, and sIGF2R levels in first-trimester maternal serum did not differ significantly between the groups (Fig. 1, C1-C4).

### Correlations between variables

**Figure 3:**
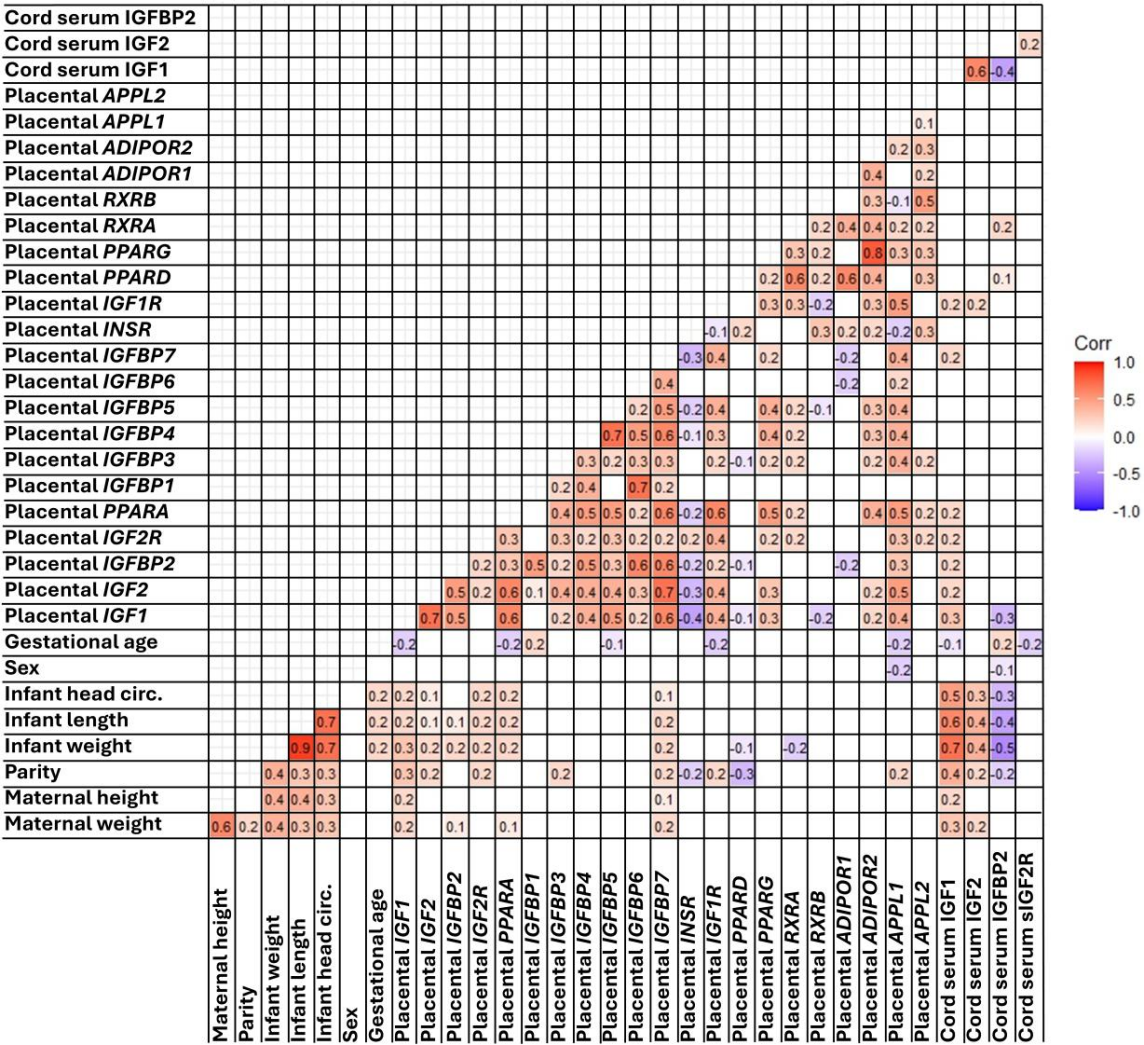
Correlation heatmap depicting statistically significant correlations between placental tissue gene expression, cord blood protein levels, and infant characteristics. Correlation coefficients are reported. The small panel to the right of the plot shows the color key for the correlation coefficients. Positive correlations are indicated in shades of red, whereas negative correlations are indicated in shades of blue. Correlations without statistical significance are not shown.

Moderate to strong positive correlations were observed between gene expression levels of *IGF1* and *IGF2* (r_s_ = 0.62, *P* < .001), *IGF1* and *IGFBP2* (r_s_ = 0.47, *P* < .001), *IGF1* and *PPARA* (r_s_ = 0.64, *P* < .001), *IGF2* and *IGFBP2* (r_s_ = 0.49, *P* < .001), and *IGF2* and *PPARA* (r_s_ = 0.62 *P* < .001). Weak to very weak correlations were observed between *IGFBP2* and *PPARA* (r_s_ = 0.35, *P* < .001), *IGF2R* and *IGF2* (r_s_ = 0.21, *P* = .002), and *IGF2R* and IGFBP2 (r_s_ = 0.19, *P* = .004). A strong positive correlation was observed between cord blood serum concentrations of IGF1 and IGF2 (r_s_ = 0.61, *P* < .001). Cord serum levels of IGF1 and IGFBP2 were inversely correlated (r_s_ = −0.51, *P* < .001) (Fig. 3).

The expression of the 5 DE genes together accounted for 6%, the expression of all 21 genes together accounted for 8%, and the expression of the 21 genes and clinical variables (sex, gestational age, maternal weight, height, parity) together explained 33% of the variation in birth weight, according to adjusted R-squared values derived from multivariable linear regression analyses. Cord blood protein levels explained 53% of the variation in birth weight. Similar results were found for birth length and head circumference (Table 3).

**Table 3:** Multiple linear regression analyses using birth weight, birth length, and head circumference as dependent variables

	Dependent variables in separate regression analyses		
Independent variables included in different models	Birth weight	Birth length	Head circumference
Placental differentially expressed genes between study groups *(IGF1, IGF2, IGFBP2, IGF2R, PPARA*)	0.058	0.042	0.028
Placental expression values of all 21 genes	0.082	0.119	0.009
Placental expression of all 21 genes and clinical characteristics	0.328	0.315	0.253
Cord blood protein concentrations (IGF1, IGF2, IGFBP2 and sIGF2R)	0.526	0.360	0.207
Cord blood protein concentrations and clinical characteristics	0.687	0.609	0.426
Placental expression of all 21 genes, cord blood protein concentrations, and clinical characteristics	0.685	0.601	0.423

Adjusted R^2^ values are reported. Clinical characteristics included sex, gestational age, and maternal weight, height, and parity.

## Discussion

In this translational study, we examined the gene and protein expression of mediators associated with the insulin/IGF, adiponectin, and PPAR signaling pathways in the human placenta in relation to birth weight. After studying the expression of a broad gene panel, we focused on the DE mediators, namely, IGF1, IGF2, IGFBP2, IGF2R, and PPARα. Their placental protein expression and serum levels in cord and maternal blood were evaluated using IHC and ELISA, respectively, to assess all compartments of interest in regard to fetal growth.

The insulin/IGF system has been extensively studied in the context of fetal growth abnormalities. Positive correlations between placental IGF1 and IGF2 gene and protein expression and infant birth weight were found in the present study, which is consistent with previous findings [23, 36]. Additionally, similar relationships were observed between cord blood levels of IGF1 and IGF2 and both birth weight and length (Fig. 3), as previously reported [23, 37].

Unexpectedly, we observed discrepancies between the IGFBP2 gene and protein expression in the placenta. The mRNA expression of *IGFBP2* was lower in the SGA and AGA groups than in the LGA group, even though the differences were small. In contrast, the protein expressions of IGFBP2 in placental tissues and in cord blood samples were greater in the SGA and AGA groups than in the LGA group. With respect to these findings, it is important to note that, due to various posttranscriptional regulatory mechanisms, one should not expect a straightforward correlation between mRNA levels and protein abundance [38]. As stated, this raises the question of whether an unmeasured regulatory factor is present. Placental pregnancy-associated plasma protein-A (PAPP-A) has attracted significant interest due to its role in regulating biologically active IGF by cleaving IGFBP-2, IGFBP-4, and IGFBP-5 [37, 39]. Whether a differential expression of PAPP-A in our study groups or any other known regulator of IGFBP2 could explain the observed discrepancies in this study remains to be determined.

Previous studies have shown a negative correlation between birth weight and placental IGFBP2 gene and protein expression, as well as cord blood protein levels [1, 40], likely due to IGFBP2's binding capacity; this results in higher levels of free IGF1 and IGF2, which stimulate growth when IGFBP2 levels are low. In addition, a negative correlation was observed between cord blood IGFBP2 levels and birth weight (Fig. 3), further corroborating the hypothesis that increased IGFBP2 expression may limit growth. These findings make the observation of lower *IGFBP2* gene expression in the context of lower birth weight contradictory. A possible explanation may lie in the reduced PPARA gene and protein expression in the placentas of SGA infants compared to those of LGA infants, and weak positive correlation between *IGFBP2* and *PPARA* gene expression, observed in this study. PPARα has been shown to stimulate *IGFBP2* gene expression [41]. This provides a plausible explanation for lower *IGFBP2* gene expression in the SGA and AGA groups compared with the LGA group; however, further investigation would be required to establish a causal relationship.

IGF2R has previously been described as a growth inhibitor [1]. However, we observed lower *IGF2R* gene expression in the SGA group than in the AGA group. In addition, weaker IGF2R staining was observed in the SGA and AGA groups than in the LGA group. The downregulation of IGF2R expression could increase IGF2 levels, potentially contributing to increased growth rather than poor growth. These findings suggest that reduced IGF2R expression in SGA infants may represent a compensatory mechanism to mitigate impaired fetal growth. These findings contrast with our findings concerning placental and cord blood protein concentrations of IGF1, IGF2, and IGFBP2, for which the variations across the 3 study groups support a stimulatory role for fetal growth. In addition, strong correlations were observed between placental gene expression and cord blood protein levels of IGF1, IGF2, and IGFBP2, suggesting a common upstream regulator, such as the mother's nutritional state [36]. In line with this suggestion, maternal weight and BMI were lowest in the SGA group and highest in the LGA group (Table 1). Furthermore, there was no apparent association between placental *IGF2R* gene expression and *IGF1*, *IGF2*, and I*GFBP2* gene expression, indicating the presence of a distinct upstream regulatory mechanism for *IGF2R* that is separate from the regulator potentially shared among the *IGF1*, *IGF2*, and *IGFBP2* genes in the placenta.

The results of murine studies suggest that PPARα deficiency is associated with miscarriage and neonatal mortality [42]. The present study is the first to report lower placental *PPARA* gene and protein expression in SGA infants than in LGA infants. In addition, *PPARA* expression was positively associated with several members of the insulin/IGF system, as well as *PPARG*. Together with the proposed regulatory action in highly metabolically active tissues and in placental lipid transfer and homeostasis [43], human placental PPARα expression may be linked to the maternal nutritional state in a similar way as the insulin/IGF system and may consequently affect fetal growth. Further investigations are needed to clarify the effects of PPARs on fetal and placental development.

Although adiponectin is reportedly expressed in the full-term human placenta, other studies have failed to confirm this [44]. In this study, no detectable adiponectin-encoding mRNA (*ADIPOQ*) expression was found, indicating that the placenta at term is not a site of adiponectin production. Discrepancies between reported findings may be due to several causes, including maternal blood contamination of placental samples and sampling variability. In addition, no association was found between infant birth weight and placental gene expression of adiponectin receptors (*ADIPOR1* and *ADIPOR2*) or their downstream effectors (*APPL1* and *APPL2*) in the present study.

Previous studies have indicated a correlation between first-trimester crown–rump length and birth weight [45]. Evaluation of maternal blood during pregnancy for potential diseases in the offspring, such as trisomy, neural tube defects, and abdominal wall defects, is considered standard medical practice [46]. In this study, no significant associations were found between IGF1, IGF2, IGFBP2, or sIGF2R levels in first-trimester maternal blood and birth weight, indicating that these levels are not predictive of birth weight. However, Åsvold et al [47] reported that increased maternal IGF1 and IGFBP1 expressions in the first and second trimesters were associated with birth weight. These findings suggest that these proteins may be useful as predictive biomarkers of fetal growth if measured repeatedly.

Linear regression analyses revealed the effects of placental gene expression, cord blood protein levels, and clinical characteristics on birth size. The ability of cord blood protein levels to predict infant weight and length was much greater than that of placental gene expression, indicating that compared with placental transcripts, cord blood protein levels more closely reflect fetal physiology. The capacity of the studied variables to explain the variation in head circumference was lower than that of birth weight and length, irrespective of the type of model used, which is in line with the unique growth of the skull [48].

The main strengths of this study include the large study population; the multilayered analysis of placental tissues, cord blood, and maternal blood; the inclusion of mediators associated with several pathways; and the careful description of clinical variables. However, certain limitations warrant further discussion. Maternal first-trimester serum samples were stored at −20 °C, a temperature that is suboptimal for protein preservation. The lack of ultrasound verification of fetal growth increases the risk of studying physiologically small infants. However, according to the study's definition of SGA, only approximately 2% of the infants were eligible for inclusion in the SGA group. Obtaining tissue samples after parturition to study the potential effects of prenatal exposure is problematic because biochemical processes in the placenta change throughout pregnancy [49]. Placental tissue is characterized by a diverse and uneven distribution of cells, and previous studies have shown that gene expression varies by location [50, 51]. To address this challenge, mRNA can be extracted from multiple sites within each placenta [51]. However, in the present study, mRNA was extracted from only 1 location, which might have influenced the results. Protein expression and localization were assessed by IHC, although an additional quantitative method, such as Western blotting, might have been valuable. Finally, it is essential to emphasize that while placental DE genes and proteins may influence cellular growth and proliferation in that tissue, these findings do not necessarily reflect changes within fetal tissues themselves.

In conclusion, the signaling pathways of placental IGF1, IGF2, and IGFBP2 are associated with increased fetal growth. Expressions of these factors show significant correlations, suggesting a potential common upstream regulator, such as nutrition. In contrast, placental IGF2R signaling is associated with decreased fetal growth, indicating a counteracting role in instances of both poor and exaggerated fetal growth. Lower levels of PPARα in the placentas of SGA infants were observed for the first time. Further studies are needed to verify our findings in other populations and to investigate how these changes affect fetal growth under various conditions.

## Data Availability

Some or all datasets generated during and/or analyzed during the current study are not publicly available but are available from the corresponding author on reasonable request.
